# Cannabis products and trends in a cohort of young adults: The VapeScan longitudinal study

**DOI:** 10.18332/tid/210320

**Published:** 2025-12-16

**Authors:** Katlyn E. McGraw, Elizabeth C. Oelsner, Nancy J. LoIacono, Siyue Gao, William Anderson, Dona Sangapalaarachchi, Vesna Illievski, Justin Liu, Silvia Martins, Tiffany R. Sanchez, Daichi Shimbo, Ana Navas-Acien

**Affiliations:** 1 Department of Environmental Health Sciences, Columbia University Mailman School of Public Health, New York, United States; 2 Department of Medicine, Columbia University Irving Medical Center, New York, United States; 3 Department of Epidemiology, Columbia University Mailman School of Public Health, New York, United States

**Keywords:** cannabis, vaping, e-cigarettes, marijuana, longitudinal

## Abstract

**INTRODUCTION:**

Cannabis is the third most used drug in the world with emerging legalization in the US and other countries. In a descriptive analysis, we report trends in cannabis use and types of products used in the VapeScan longitudinal study, a cohort study of young adults designed to investigate subclinical health effects of e-cigarette use.

**METHODS:**

The VapeScan study recruited 372 adults aged 18–50 years in the New York City area in 2021–2024, independently of cannabis use. At Visit 1, we asked about cannabis use, and at Visit 2, we implemented a more detailed questionnaire to characterize longitudinal trends and methods of cannabis use. We performed descriptive analyses to compare and report cannabis and tobacco use among participants groups of: 1) no substance use, 2) exclusive cannabis use, 3) exclusive e-cigarette use, and 4) dual substance use (e-cigarette and cannabis use).

**RESULTS:**

Participants had a median age of 26 years (IQR: 21–33), were 50.5% male, 48.1% female, and 1.3% non-binary. At Visit 1, 125 (33.6%) participants reported dual substance use, 15 (4%) reported exclusive cannabis use, 129 (34.7%) reported e-cigarette but no cannabis use, and 103 (27.7%) reported no substance use. At Visit 2, 128 of 217 (58.9%) participants reported cannabis use; 63 (29.0%) vape cannabis, 61 (28.1%) smoke cannabis, 111 (51.2%) edible cannabis, 69 (31.8%) CBD, and 8 (3.7%) topical cannabis. Frequency and intensity varied by method of use. Self-reported trends of vaped or smoked cannabis use varied between visits, with 28 (20.9%) becoming new users at Visit 2, while quitting only happened in 5 (6.3%) of those who vaped or smoked cannabis at Visit 1 (p=0.007).

**CONCLUSIONS:**

Our findings support that cannabis use is diverse and complex and is growing in urban communities, requiring further investigation to better understand use patterns and potential exposure to cannabis-related contaminants and related health effects.

## INTRODUCTION

Cannabis use in the United States represents a multifaceted and evolving public health challenge. As of 2023, 61.8 million people, or 21.8% of Americans aged ≥12 years, reported using marijuana at least once in the last year^1^. Frequent cannabis use is becoming more widespread and will likely continue to increase with shifts in social norms, policy liberalization, and greater availability and promotion of cannabis products^2^. The prevalence of cannabis use among teens and young adults is rising, along with the various forms of cannabis products used, such as edibles, concentrates, and vaporized products (vapes), adding to the complexity of studying the health effects of cannabis use^3^. Young adults often engage in polysubstance use, combining cannabis with alcohol, tobacco, or other drugs, further complicating research efforts to disentangle health effects of cannabis use from other substances^4,5^.

The VapeScan study is an observational longitudinal cohort study that recruited participants in the New York City (NYC) area from May 2021 to May 2024. Since legal recreational cannabis sales began in NYC on 29 December 2022 and in New Jersey on 21 April 2022, the VapeScan study provides an opportunity to explore current trends and patterns of cannabis use in a contemporary cohort of young adults, including its frequency and types of products, as well as polysubstance use, in a cohort of young adults based in the NYC area.

## METHODS

### Study population

The VapeScan study is a longitudinal cohort study of subclinical cardiopulmonary disease among diverse young adults aged 18–50 years, in the New York area. Participants were recruited using flyers, social media campaigns, word of mouth, vape lounges/ shops in New York and neighboring New Jersey, and RecruitMe, a Columbia-based online recruitment platform. Participants were assessed three times: Visit 1 at baseline, Visit 2 approximately 12 months after the initial examination, and Visit 3 approximately 24 months after the initial examination. As of December 2024, 1633 participants were screened, 547 participants gave informed consent, 414 completed the questionnaire, 372 completed the first examination visit, and 217 completed the second examination visit (as of April 2025, the 2nd and 3rd examinations are ongoing). Participants who met enrollment criteria were invited to participate and provided written informed consent. The Institutional Review Board at Columbia University approved the study (Approval number: AAAS9701).

### VapeScan e-cigarette and vape device use categorization

At Visit 1, VapeScan participants were recruited into one of four categories based on self-report: 1) control (never use of e-cigarettes or combustible cigarettes), 2) current use of e-cigarettes and never use of combustible cigarettes, 3) current use of e-cigarettes and former use of combustible cigarettes, and 4) dual use of e-cigarettes and combustible cigarettes. We classified participants who reported using e-cigarettes some days or every day and smoked fewer than 100 cigarettes in their lifetime as current use of e-cigarettes and never use of combustible cigarettes. Those who reported using e-cigarettes some days or every day and smoking at least 100 cigarettes in their lifetime were classified as current use of e-cigarettes and former use of combustible cigarettes. Dual users were those who reported using e-cigarettes every day or some days and smoking cigarettes every day or some days.

### Cannabis and nicotine use categorization

The baseline questionnaire of the VapeScan study included questions about cannabis use. At the baseline visit, participants were asked: ‘Have you ever smoked marijuana or hashish regularly (at least once per month)?’ and ‘Do you smoke marijuana or hashish regularly now?’ to determine ever and current smoked cannabis use. Participants were additionally asked: ‘Have you ever used marijuana, marijuana concentrates, marijuana waxes, THC, or hash oils in a vaping device?’ and ‘Do you now use vaping products to consume marijuana, marijuana concentrates, marijuana waxes, THC, or hash oils?’ to determine current vaped cannabis use. If participants responded ‘yes’ to either question regarding currently smoking or vaping cannabis, they were categorized as current cannabis users. Participants who responded ‘yes’ to ever smoking or vaping cannabis, but no to current smoking or vaping cannabis were categorized as ever cannabis users. Participants were categorized as current e-cigarette users if they self-reported using e-cigarettes every day or some days. Based on self-reported current cannabis use and e-cigarette use, we further categorized individuals into four types of cannabis users at baseline: 1) control – no cannabis use, no e-cigarette use, no combustible cigarette use (no substance use for simplicity); 2) exclusive cannabis use – smoking or vaping cannabis only; 3) exclusive e-cigarette use, current e-cigarette use and no current cannabis use; and 4) dual substance use – current e-cigarette and current cannabis use.

### Extended cannabis questionnaire

At Visit 2, we asked: ‘Have you used vaping products to consume marijuana, marijuana concentrates, marijuana waxes, THC, or hash oils since your last visit?’ and ‘Have you smoked marijuana or hashish since the last visit?’ to determine consistency of use since the last visit. At Visit 2, we expanded the cannabis use questionnaire to include questions on additional types and methods of cannabis use (dry leaf, vape, topical, edible), the active cannabinoid tetrahydrocannabinol (THC) or cannabidiol (CBD), frequency of use, primary reasons for use, and history of use. Questions were modeled after existing cannabis use surveys^6,7^.

### Covariates

Age, gender identity, race, ethnicity, and education level were acquired from self-reported questionnaires at baseline. Race and ethnicity were classified as White, Black, Asian, Native American, more than one race, or no response; and participants self-reported Hispanic or Latino as ‘yes’ or ‘no’. We reclassified education level as a high school education or lower, some college, Bachelor’s degree, graduate degree, or no response.

### Statistical analysis

Participants were categorized into four groups of cannabis and e-cigarette use groups at baseline: control (no substance use), exclusive current cannabis use, exclusive current e-cigarette use, and dual current cannabis and e-cigarette use, as previously described. We conducted descriptive analyses overall and by cannabis and e-cigarette use groups using the median and interquartile range (IQR) for continuous variables, and frequency and percentage for categorical variables. Among the participants with data available at Visit 2, we further described consistency of cannabis use, primary modes of cannabis consumption (edibles, topical use, and CBD), and frequency of use. To compare medians among continuous variables and compare groups among categorical variables, we used the Kruskal-Wallis and χ^2^ test or Fisher’s exact test, respectively. To evaluate the stability of self-reported vaping and smoking cannabis across Visits 1 and 2, we used frequency tables and χ^2^ to assess trends between participants who self-reported currently vaping or currently smoking cannabis at Visit 1, and participants who self-reported vaping cannabis or smoking cannabis since the last visit at Visit 2. All statistical analyses were performed using R software (version 4.4.1) and a two-tailed p<0.05 was considered statistically significant.

## RESULTS

Demographic characteristics of the sample are described in Table 1. At baseline, 372 participants with median age 26 years (IQR: 21–33), were 50.5% male, 48.1% female, and 1.3% non-binary individuals. Participants self-reported race as 46.8% White, 15.3% Black, 19.9% Asian, 2.2% Native American, 5.6% as more than one race, and ethnicity as 29.3% Hispanic or Latino. Most participants had some college education (33.1%) or already held a higher education degree (53.5%). Overall, 140 (37.6%) VapeScan participants reported currently using smoked or vaped cannabis at baseline. After grouping individuals by use type, 125 (33.6%) reported dual cannabis and e-cigarette use, 15 (4%) reported exclusive cannabis use, 129 (34.7%) reported e-cigarette but no cannabis use, and 103 (27.7%) reported no substance use (control). Participants reporting dual cannabis and e-cigarette use were younger, of median age 25 years (IQR: 21–31), than all other use groups, although age differences among the 4 groups were not statistically significant. Frequency of dual cannabis and e-cigarette use was similar among men and women, and was reported by 4 of 5 non-binary participants. Participants in the no substance use group were less likely have an education level of high school or lower, and more likely to be in the graduate school category compared to the cannabis and e-cigarette groups (p<0.001).

**Table 1:** VapeScan participant characteristics at Visit 1 (n=372), and extended cannabis questionnaire at Visit 2 (n=217), overall and by use group

*Characteristics*	*Overall* *n (%)*	*No substance* *use (control)* *n (%)*	*Cannabis use* *only* *n (%)*	*E-cigarette use* *only ^a^* *n (%)*	*Dual* *e-cigarette and* *cannabis use ^b^* *n (%)*	p*
**Visit 1**						
**Total,** n	372	103	15	129	125	
**Age** (years), median (IQR)	26 (21–33)	26 (22–33)	28 (20.5–31)	26 (21–34)	25 (21–31)	0.71
**Gender**						0.44
Male	188 (50.5)	54 (52.4)	9 (60.0)	65 (50.4)	60 (48.0)	
Female	179 (48.1)	49 (47.6)	6 (40.0)	63 (48.8)	61 (48.8)	
Non-binary	5 (1.3)	0 (0)	0 (0)	1 (0.8)	4 (3.2)	
**Race**						0.06
White	174 (46.8)	51 (49.5)	7 (46.7)	59 (45.7)	57 (45.6)	
Black	57 (15.3)	16 (15.5)	4 (26.7)	16 (12.4)	21 (16.8)	
Asian	74 (19.9)	25 (24.3)	1 (6.7)	32 (24.8)	16 (12.8)	
Native American	8 (2.2)	2 (1.9)	0 (0)	1 (0.8)	5 (4.0)	
More than one race	21 (5.6)	3 (2.9)	2 (13.3)	4 (3.1)	12 (9.6)	
No response	38 (10.2)	6 (5.8)	1 (6.7)	17 (13.2)	14 (11.2)	
**Hispanic**						0.94
Yes	109 (29.3)	32 (31.1)	5 (33.3)	36 (27.9)	36 (28.8)	
No	263 (70.7)	71 (68.9)	10 (66.7)	93 (72.1)	89 (71.2)	
**Education level**						0.003
High school or lower	46 (12.4)	5 (4.9)	2 (13.3)	18 (14.0)	21 (16.8)	
Some college	123 (33.1)	22 (21.4)	7 (46.7)	47 (36.4)	47 (37.6)	
Bachelor’s	133 (35.8)	44 (42.7)	6 (40.0)	42 (32.6)	41 (32.8)	
Graduate	66 (17.7)	31 (30.1)	0 (0)	20 (15.5)	15 (12.0)	
No response	4 (1.1)	1 (1.0)	0 (0)	2 (1.6)	1 (0.8)	
**Ever use cannabis**						<0.001
Yes	262 (70.4)	39 (37.9)	15 (100)	83 (64.3)	125 (100)	
No	110 (29.6)	64 (62.1)	0 (0)	46 (35.7)	0 (0)	
**Cannabis use status**						<0.001
Current	140 (37.6)	0 (0)	15 (100)	0 (0)	125 (100)	
Former	122 (32.8)	39 (37.9)	0 (0)	83 (64.3)	0 (0)	
Never	110 (29.6)	64 (62.1)	0 (0)	46 (35.7)	0 (0)	
**Smoking status**						<0.001
Current	93 (25.0)	0 (0)	0 (0)	43 (33.3)	50 (40.0)	
Former	66 (17.7)	0 (0)	0 (0)	37 (28.7)	29 (23.2)	
Never	213 (57.3)	103 (100)	15 (100)	49 (38.0)	46 (36.8)	
**Visit 2**						
**Total,** n	217	76	7	61	73	
**THC edibles**						<0.001
Yes	111 (51.2)	25 (32.9)	6 (85.7)	22 (36.1)	58 (79.5)	
No	97 (44.7)	48 (63.2)	1 (14.3)	34 (55.7)	14 (19.2)	
Missing	9 (4.1)	3 (3.9)	0 (0)	5 (8.2)	1 (1.3)	
**Days THC edibles used in the last month**						0.002
≤10	35 (31.5)	7 (28.0)	2 (33.3)	5 (22.7)	21 (36.2)	
>10	5 (4.5)	1 (4.0)	0 (0)	0 (0)	4 (5.0)	
**Edibles unit dose** (mg THC)						0.001
≤10	38 (34.2)	13 (52.0)	3 (50.0)	5 (22.7)	17 (29.3)	
>10	24 (21.6)	2 (8.0)	0 (0)	7 (31.8)	15 (25.9)	
**CBD cannabis**						0.22
Yes	69 (31.8)	20 (26.3)	3 (42.9)	15 (24.6)	31 (42.5)	
No	141 (65.0)	55 (72.4)	4 (57.1)	42 (68.9)	40 (54.8)	
**CBD route**						0.11
Inhaled	5 (7.2)	0 (0)	1 (33.3)	0 (0)	4 (5.5)	
Edible	22 (31.9)	6 (30.0)	0 (0)	6 (40.0)	10 (32.3)	
Topical	7 (10.1)	3 (15.0)	0 (0)	3 (20.0)	1 (3.2)	
Other	2 (2.9)	1 (5.0)	0 (0)	0 (0)	1 (3.2)	
**Topical THC**						0.61
Yes	8 (3.7)	2 (2.6)	0 (0)	2 (3.3)	4 (5.5)	
No	201 (92.6)	73 (96.1)	7 (100)	55 (90.2)	66 (90.4)	

a Participants who self-reported currently using e-cigarettes every day or some days but reported they do not currently use cannabis every day or some days.

b Participants who self-reported currently using e-cigarettes and cannabis every day or some days.

* Values were derived using Kruskal-Wallis tests to test the difference in means among groups of continuous variables, and the Fisher exact test to test the difference among categorical variables. IQR: interquartile range. THC: tetrahydrocannabinol. CBD: cannabidiol.

At Visit 2, 217 participants (58.3%) had completed the expanded cannabis questionnaire (Table 2). Comparing those 217 participants across visits, the percentage of participants who self-reported vaping cannabis changed from 29.0% at Visit 1 to 35.9%, which was not statistically significant (p=0.10), while for smoking cannabis, it changed from 28.1% to 43.8% (p=0.001), and for either vaping or smoking cannabis it changed from 36.9% to 47.5% (p=0.023). Among the 80 participants vaping or smoking cannabis at Visit 1, a total of 5 (6.3%) had quit by Visit 2 while among the 137 participants not smoking or vaping cannabis at Visit 1, 28 (20.9%) had now started to vape or smoke cannabis at Visit 2 (p=0.007).

**Table 2:** Cannabis use (vaping or smoking) at Visits 1 and 2 among VapeScan participants who returned for Visit 2 (N=217)

	*Visit 1* *n (%)*	*Visit 2* *n (%)*	p*
**Total,** n	217	217	
**Vape cannabis^a^**			0.1
Yes	63 (29.0)	78 (35.9)	
No	154 (71.0)	136 (62.7)	
**Smoke cannabis^b^**			0.001
Yes	61 (28.1)	95 (43.8)	
No	156 (71.9)	119 (54.8)	
**Vape or smoke cannabis^c^**			
Yes	80 (36.9)	103 (47.5)	0.023
No	137 (63.1)	111 (51.2)	
NA	0 (0)	3 (1.38)	

a For vape cannabis use between visits, we compared responses from Visit 1 item ‘Do you now use vaping products to consume marijuana, marijuana concentrates, marijuana waxes, THC, or hash oils?’ to Visit 2 item ‘Have you used vaping products to consume marijuana, marijuana concentrates, marijuana waxes, THC, or hash oils since your last visit?’.

b For smoke cannabis use between visits, we compared responses from Visit 1 item ‘Do you smoke marijuana or hashish regularly now?’ to Visit 2 item ‘Have you smoked marijuana or hashish since the last visit?’.

c For vape or smoke cannabis use between visits, we compared if participants responded ‘yes’ for either vape or smoke cannabis use at each visit.

* Significance of χ^2^ tests between visits.

**Figure 1:**
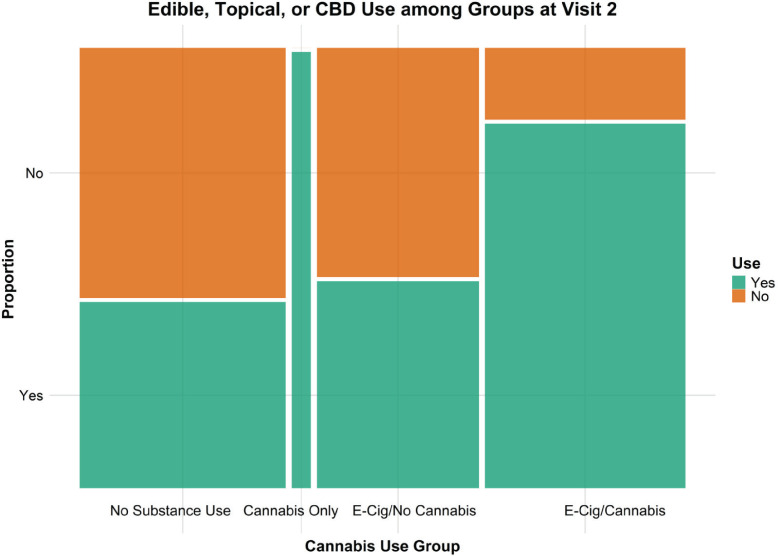
Proportion of VapeScan participants at Visit 2 (n=217) that use cannabis in forms other than smoking or vaping (edibles, topicals, or CBD), by use group: control (no substance use), current cannabis use, current e-cigarette use, and dual use

Among 76 participants in the no-substance use group (i.e. reporting neither vaping or smoking cannabis containing THC or tobacco at baseline) who attended Visit 2 (Table 1), 32 (42%) participants reported using other forms of cannabis at Visit 2 (Figure 1). Because other forms of cannabis were not asked at Visit 1, we do not know if this is a de novo uptake or these other forms of cannabis were already in use at Visit 1. Among participants who used cannabis, 63 (29.0%) reported regularly vaping cannabis, 61 (28.1%) reported regularly smoking cannabis, 111 (51.2%) reported using edible cannabis products, 69 (31.8%) reported using CBD, and 8 (3.7%) reported using topical cannabis products. Of the 95 participants who reported using edible cannabis products, 38 (34.2%) reported using unit doses of ≤10 mg THC and 24 (21.6%) report using >10 mg THC. Of the 95 participants who reported using edible cannabis products, 35 (31.5%) reported consuming edibles between 1 and 10 days in the last month and 5 (4.5%) reported consuming edibles >10 days in the last month. Of the 69 participants who reported using CBD, 23 were classified as no-substance use at visit as they were not vaping or smoking cannabis at the time. The most reported route of CBD use was edibles (31.9%) followed by topical application (10.1%) and inhalation (7.2%).

## DISCUSSION

In this descriptive longitudinal study of current cannabis use among young adults in the NYC area, we found that self-reported trends of cannabis use (vaped or smoked) varied between visits, particularly with 20.9% becoming new users at Visit 2, while quitting only happened in 6.3% of those who vaped or smoked cannabis at Visit 1. Additionally, we found that young adults who do not vape or smoke cannabis may still use other forms of cannabis such as consumables, topicals, or CBD. Furthermore, polysubstance use of cannabis products, along with nicotine and tobacco products is common among young adults who currently use cannabis. Cannabis is emerging as a major public health concern that has, for too long, been overlooked. As the third most widely used drug worldwide, it is essential to recognize its prevalence and understand the potential health implications^8^. Our study highlights the importance of studying cannabis exposure, as it can serve as a source of exposure to several environmental contaminants beyond the potential health effects of the cannabinoids^9^. Our findings support that cannabis use is diverse and complex and requires further investigation to better understand use patterns and potential exposure to cannabis-related contaminants^10^.

According to the 2024 National Survey on Drug use and Health, marijuana use in the past year increased from 19% in 2021 to 22.3% in 2024 among people aged ≥12 years. Among people aged ≥26 years, marijuana use increased from 17.3% to 21.7% over the same period^11^. However, from 2021 to 2024, there was no observed change in the percentage of people who initiated marijuana use among people aged ≥12 years. Contrary to national data, VapeScan participants showed a higher rate of cannabis initiation at Visit 2. Specifically, a greater proportion of participants initiated cannabis use compared to those who stopped using it at the same visit. New marijuana use among VapeScan participants may have been influenced by the timing of legal recreational marijuana sales, which began in NYC and New Jersey in 2022 during the study’s recruitment and follow-up. It is possible that our numbers are relevant for individuals who use e-cigarettes or are interested in e-cigarette research, and do not necessarily reflect rates of initiation in the general population of young adults in NYC. Data on specific rates of initiation in NYC and New York state are not available^12^. More research is needed to clarify how access and legalization influence patterns of cannabis use.

Cannabis can be consumed in multiple ways including smoking, vaping, and ingesting it through food or beverages. According to the 2024 National Survey on Drug use and Health, among people aged ≥12 years who reported using marijuana in the last year or the last month, 73.9% smoked marijuana, 49.8% ate or drank marijuana, and 39.8% vaped marijuana^11^. We observed similar trends among VapeScan participants, with smoking, edibles, and vaping as the most common modes of use. Cannabidiol (CBD) use is not categorized as a method of cannabis use in the national survey since federally legal CBD products must contain <0.3% tetrahydrocannabinol (THC), the psychoactive component of cannabis. Some VapeScan participants who abstained from THC or nicotine use still reported using CBD. CBD’s popularity, driven by its potential health benefits, has created a complex landscape where individuals seek alternatives that do not have the psychoactive effects of THC^13^. However, because CBD products are not regulated by the USDA or FDA in the same way as state-regulated THC products^14^, these findings highlight the importance of considering the broader range of cannabis compounds that individuals may encounter.

A relevant observation from our study is the number of individuals that concurrently vape or smoke cannabis while also using consumable cannabis products such as edibles, including participants who were initially classified in the no substance use category as they did not report vaped or smoked cannabis. This underscores the multifaceted nature of cannabis use and the potential for varied exposure profiles. Understanding the co-occurrence of different consumption methods raises questions about the cumulative effects of these diverse exposure pathways. Our study supports that additional epidemiological research, specifically on cannabis use, is critically needed, including longitudinal studies to track changes in use patterns over time and investigations into the long-term health outcomes associated with different use profiles.

To address this challenge, innovative methodologies and ongoing monitoring are essential to capture the dynamic nature of cannabis exposure. The intersection of regulatory policies, product innovation, and evolving consumer preferences adds to the complexities of effectively assessing cannabis exposure. Given that cannabis use remains illegal at the federal level^15^ and is relatively understudied in an industry experiencing rapid growth, there is a pressing need to understand and identify contemporary trends and methods of use.

### Limitations

Although these descriptive study findings highlight new and emerging trends among young adults who use cannabis, limitations persist. All findings were derived from self-reported questionnaire data and may be prone to recall bias, information bias, and misclassification. Additionally, this cohort was established for a study of e-cigarette use and there may be some selection bias from targeted recruitment not originally designed to measure cannabis use and also not-representative of young adults in NYC. The findings may not be generalizable to other states or other countries as legalization and acceptance and prevalence of recreational use varies within the US and globally. Data from other cohorts designed to study cannabis use and simultaneous nicotine use, like the Population Assessment of Tobacco and Health (PATH) or expanded questionnaires in the National Health and Nutrition Examination Survey (NHANES), may help elucidate trends in a larger sample size.

## CONCLUSIONS

Our study highlights the importance of a comprehensive approach to characterizing cannabis exposure. This includes considering the wide array of cannabis-derived products, accounting for multiple modes of consumption, and addressing the ongoing challenge of assessing real-world exposure. A deeper understanding of these complexities is essential for advancing research on the potential harms of cannabis use and the factors that influence them. Such evidence will ultimately provide a stronger foundation for guiding public health responses and informing future policy discussions.

## Data Availability

The data supporting this research cannot be made available for privacy or other reasons. Protected health information data were used.
